# Development and Characterization of Near-Infrared Detectable Twin Dye Patterns on Polyester Packaging for Smart Optical Tagging

**DOI:** 10.3390/polym17202784

**Published:** 2025-10-17

**Authors:** Silvio Plehati, Aleksandra Bernašek Petrinec, Tomislav Bogović, Jana Žiljak Gršić

**Affiliations:** Department of Informatics & Computing, Zagreb University of Applied Sciences, 10000 Zagreb, Croatia; abernasek@tvz.hr (A.B.P.); tbogovic@tvz.hr (T.B.); jana@tvz.hr (J.Ž.G.)

**Keywords:** near-infrared (NIR), twin dyes, polyester textiles, NIR absorbance, spectroscopy, BPW83 sensor, NIR light emitting diodes

## Abstract

Smart polyester materials with embedded near-infrared (NIR) functionalities offer a promising pathway for low-cost, covert tagging, and object identification. In this study we present the development and characterization of polyester packaging surfaces printed with spectrally matched twin dyes that are invisible under visible light but selectively absorbed in the NIR region. The dye patterns were applied using a Direct-to-Film transfer (DTF) method onto polyester substrates. To validate their optical behavior, we applied a dual measurement approach. Laboratory grade NIR absorbance spectroscopy was used to characterize the spectral profiles of the twin dyes in the 400–900 nm range. A custom photodiode-based detection system was constructed to evaluate the feasibility of low-cost, embedded NIR absorbance sensing. Results from both methods show correlation in absorbance contrast between the dye pairs, confirming their suitability for spectral tagging. The developed materials were evaluated in a real-world detection scenario using commercially available NIR cameras. Under dark field conditions with edge illuminated planar lighting, the twin dye patterns were successfully recognized through custom software, enabling non-contact identification and spatial localization of the NIR codes. This work presents a low-cost, scalable approach for smart packaging applications based on optical detection of actively illuminated twin dyes using accessible NIR imaging systems.

## 1. Introduction

The development of smart textile wearables for the identification and tracking [1] of objects in demanding conditions, such as at night, represents one of the key research areas in the fields of security [2], robotics, computer vision, and functional materials [3]. The need for reliable systems capable of identifying objects in complete darkness using the NIR spectrum instead of thermal signatures is increasingly prominent in SAR (Search and Rescue) operations, as well as in military and civilian applications. Such systems must enable fast and accurate object detection from the air (via drones or helicopters) [4].

Currently available technologies in this field possess important but limited features: thermal cameras detect bodies based on heat emission, but their efficiency drops when body temperature matches the environment (e.g., hypothermia) or when the person is hidden [5]. IR reflective markers require an external radiation source and a specific illumination angle for effective reflection [6]. Visual markers in the visible spectrum are easily detectable but do not work in darkness and visually compromise the design of the equipment. Given these limitations, a new hybrid system had to be developed that combines invisible active identification through spectrally specific markers and energy efficient illumination using the NIR spectrum. In contrast to conventional reflective IR tags, which rely on external illumination and precise angular reflection, the proposed system integrates active NIR illumination directly into the wearable, ensuring reliable detection regardless of orientation or ambient lighting. Unlike thermal imaging systems that depend on heat signatures, our approach is unaffected by temperature equalization or occlusion and maintains functionality even when thermal contrast is lost. Compared with other optical tracking technologies, the presented NIR-based hybrid system provides a balanced solution between visibility, energy efficiency, and spectral selectivity. This combination of active NIR illumination, spectrally coded materials, and real-time computer vision processing extends the current state of the art in object identification under low-light or no-light conditions.

This paper presents a system of NIR illuminated smart bags with visually coded markers made from polyester materials, integrated with NIR light emitting diodes (LEDs at a wavelength of 850 nm) and passive NIR markers printed using dyes with specific absorption in the NIR spectrum. The system operates based on the optical detection of actively illuminated optical STag markers [7] using an NIR camera. The camera captures reflected radiation from IR LEDs embedded in the bag. The codes are designed to remain invisible in the visible spectrum but are clearly detectable by the camera in the NIR range. Computer vision software and algorithms OpenCV [8] with STag library detect the position and orientation and decode the data from the codes, enabling precise recognition and the tracking of tagged objects even in complete darkness.

The “twin dyes” technology used in this work is based on the concept that two visually identical samples in the visible spectrum differ in their absorption properties in the NIR range. This implementation uses the INFRAREDESIGN^®^ approach [9], in which selected dyes are formulated to show significant absorption differences between 700 and 1000 nm without altering color perception in the visible part of the spectrum. This design allows functional codes to be hidden within an aesthetically neutral surface, making it ideal for applications in smart packaging, security tagging, and the identification of people or equipment [10].

For in situ absorbance measurement, we developed a portable sensor-based system using the BPW83 infrared photodiode [11], optimized for high sensitivity near 850 nm. The sensor is connected to a Raspberry Pi Pico microcontroller [12] via a quad operational amplifier (op-amp) [13] that provides signal conditioning, enabling on-board processing of the measured data and absorbance evaluation. This approach supports users during marker fabrication without requiring expensive laboratory spectroscopy equipment. The dyes used were specifically designed for absorption in the 400–900 nm range and were characterized using spectroscopy with a forensic laboratory device [14], a portable spectrophotometer [15], and a custom sensor system based on the BPW83 photodiode.

Measurements obtained from the BPW83 sensor (Vishay Intertechnology, Inc., Malvern, PA, USA) were compared with spectroscopy results from the Projectina Docucenter 4500 (Projectina AG, Heerbrugg, Switzerland). Differences between dye twins in the visible range were assessed using the X-Rite eXact (X-Rite Inc., Grand Rapids, MI, USA) with the X-Rite eXact Manager software version 1.7 (build 3834).

The bag was produced using Backlit 190 g/m^2^ white material [16], which is classified as flame-retardant according to DIN 4102 B2 [17]. The optical tags were made from Canvas Polyester 195 g/m^2^ white matte material [18]. Canvas Polyester consists of 100% linear monoaxial filament polyester fibers with a double coating and is classified as flame-retardant according to DIN 4102 B2 [17]. This material is suitable for the heat press application of DTF (Direct-to-Film) prints onto textile. Inkjet printing was carried out using a Direct-to-Film (DTF) desktop printer [19]. The optical tags were optimized for maximum possible contrast at 850 nm, with analysis of color difference values between the twin dyes and an evaluation of code visibility detection under controlled conditions using an NIR camera. This enables multiple absorption validation: laboratory spectroscopy reference, industrial spectrophotometer colorimetry, low-cost sensor system, and real-time NIR camera detection.

A particularly important aspect of the system is the design of the markers using “spectroscopic twins” visual elements that are identical in the visible spectrum but differ in the NIR range. This allows data to be hidden in the invisible NIR domain without compromising the aesthetic appearance or impression of the bag. These markers are detected in the dark using the bag’s own IR illumination, enabling the selective identification of individuals wearing the system. This method combines materials science with functionality to create a unique security tool.

On the image processing side, a computer algorithm is used to recognize STag markers, which allow unique identification, position, and orientation determination. STag markers are compatible with open-source computer vision libraries (OpenCV). This approach makes the system suitable for mobile applications and automatic image processing via drones.

The primary objective of this study is to develop and validate a system that integrates polyester materials, dye formulation, and application on polyester substrates, the design of an optoelectronic infrared system for illumination and absorption measurement, and the automated recognition and classification of codes in the NIR range.

This work introduces a new approach to marking individuals within a group, enabling tactical tracking via spectrally selective markers that do not depend on body temperature, are invisible to the human eye, and do not require external illumination. The paper also presents a new in situ absorption measurement technique at 850 nm. The proposed marking solution is modular, low-cost, and energy-efficient, and is fully adaptable to future upgrades, including multi-spectral systems, expanded encoding schemes, additional communication modules, and newly integrated sensors. The total material cost of the prototype, including the control electronics, sensor, and optical components, is below EUR 50, which is significantly lower than that of comparable NIR detection instruments. The prototype marking system is lightweight (<350 g) and operates autonomously, requiring only a 5 V power supply and no external control unit, which makes it suitable for portable and field-deployable applications.

## 2. Materials and Methods

### 2.1. Polyester Bag Material

The prototype bag was fabricated from Guandong Texon Backlit (190 g/m^2^) white backlit material (Guandong S.p.A., Milano, Italy). Texon Backlit consists of 100% linear monoaxial polyester fibers with a vinyl emulsion and is classified as flame-retardant according to DIN 4102-B2 [17]. The material is used commercially for tents, sunshades, inflatable structures, and exhibition light boxes. For marking, the bag includes a sewn-in pocket that houses a light-emitting module, and a sheet carrying the printed STag marker is applied on top.

The marker substrate is Guandong Canvas Polyester (195 g/m^2^) white matte material (Guandong S.p.A., Milano, Italy). It consists of 100% linear, monoaxial polyester fibers with a double coating and is classified as flame-retardant according to DIN 4102-B2 [17]. The matte finish minimizes reflections on the application side.

### 2.2. AZON Pronto Printing Inks

The NIR-absorbing twin dye formulation was printed onto a Canvas Polyester substrate using a Direct-to-Film (DTF) process with an AZON Pronto printer (Azonprinter d.o.o., Zagreb, Croatia) and transferred to the fabric by heat press. The process uses CMYK + white inks, which are standard, commercially available consumables for the AZON Pronto (AZON Pronto inks). Printing was performed at a native resolution of 1440 dpi, with an estimated ink droplet volume of 8–10 pL, in two-pass mode to achieve a dry film thickness of approximately 5 µm. After printing onto the transfer film, the printed layer was placed in a pre-heating oven at 100 °C for approximately 10 s. A fine white nano-powder adhesive was then applied to the freshly printed surface, facilitating the subsequent transfer from the film to the substrate, in this case, a Guandong Canvas Polyester (195 g/m^2^, white matte) material. Following powder application, the transfer film was returned to the oven and cured at 100 °C for approximately 60 s. The prepared transfer film and canvas substrate were then laminated using a flatbed heat press. The transfer process was carried out at 120 °C for 12 s, during which the printed layer was thermally bonded to the textile substrate.

The dye formulation was defined digitally via CMYK in the RIP workflow. The absolute dry ink layer thickness on the transfer was not directly measured. However, in inkjet deposition at comparable droplet volumes and pass counts, the cured film is typically on the order of a few micrometers. In our experiments, all printer parameters (resolution, pass mode, white overprint, and curing profile) were kept constant, ensuring reproducibility of optical measurements. The STag marker size is 120 × 120 mm.

**Figure 1:**
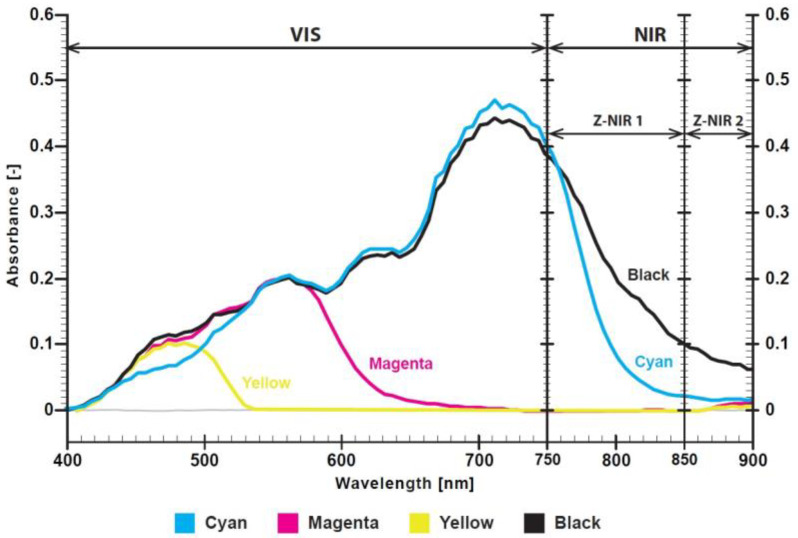
Absorbance spectra of Azon Pronto CMYK dyes (400–900 nm).

The CMYK spectra display the expected complementary behavior, where cyan absorbs in the red, magenta in the green, yellow in the blue, and black is broadband, as shown in Figure 1. Cyan dye exhibits a dominant absorption at 710 nm with a weaker shoulder near 560 nm. Cyan dye absorbance decreases toward shorter wavelengths, and in the NIR, it falls to near zero by 850 nm. Magenta dye shows an absorption maximum at 560 nm (green region), with higher transmittance in the blue and red bands. Yellow dye displays a principal maximum at 480 nm with a short-wavelength tail toward the UV. The absorbance of yellow dye above 530 nm is close to zero, yielding high transmittance in the green–red region. Black dye shows broadband absorption from 400 to 900 nm with a local maximum near 710 nm, measurable absorbance into the NIR up to 900 nm, and a weak shoulder near 560 nm.

### 2.3. NIR Illumination Setup

**Figure 2:**
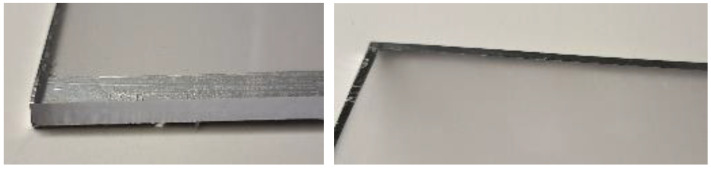
PMMA plexiglass manufactured by Gutta, prepared for experiment.

To enable planar optical illumination via total internal reflection (TIR [20]), a 150 × 200 × 4 m PMMA plexiglass light guide was prepared. The plexiglass is manufactured by Gutta [21]. The edges were polished except for one side, which was sanded to increase diffuse scattering towards the sample surface, as shown in Figure 2. The edges are sealed with aluminum tape on all sides to improve total internal reflection and left open on the side for the illumination by IR light emitting diodes (LEDs).

**Figure 3:**
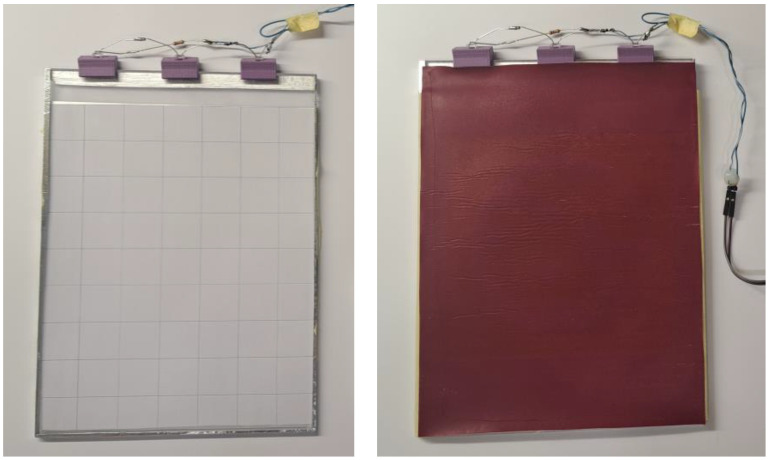
PMMA plexiglass with three IR LEDs arranged to ensure the best lighting dispersion across the printed sample (**left**) and PMMA plexiglass setup with printed sample (**right**).

NIR water clear LEDs (GaAlAs, 850 nm nominal peak [22]) manufactured by Luckylight Electronics Co., Ltd. (Shenzhen, China) were mounted along the polished edge at an angle optimized for TIR coupling (approximately 20–30° angle from the surface plane). Each LED has a typical forward voltage of 1.45 V at IF ≈ 20 mA, a radiant intensity of ~15 mW/sr at 20 mA, and a half-intensity beam angle of ~30°. Illumination was provided by three NIR LEDs connected in series with an 8.1-ohm resistor, and the forward current was measured at 18.1 mA. The LED drive current was limited to a maximum of 20 mA, ensuring stable illumination intensity while protecting the LEDs from overloading. The NIR LED illumination module consumes less than 0.1 W (approximately 81 mW at 4.5 V and 18.1 mA for three series-connected LEDs), confirming the system’s low power requirements and making it suitable for battery-powered and field-deployable applications. The LEDs were empirically arranged to ensure optimal light dispersion across the sample, as shown in Figure 3.

This illumination configuration was deliberately chosen to balance between performance, simplicity, and cost-efficiency. Using three infrared LEDs provides sufficient irradiance and spatial uniformity for reliable detection while significantly reducing system complexity and power consumption. Increasing the number of LEDs could marginally improve uniformity but would also increase cost and assembly complexity. The selected configuration therefore represents an optimal compromise for a compact, low-cost detection system.

### 2.4. Absorbance Measurements of Detectable Twin Dye Pattern

Spectral absorbance of the printed patterns on polyester substrate was measured using a laboratory-grade SP-2000 spectrometer (400–900 nm) integrated into a Projectina Docucenter 4500 forensic workstation. An additional absorbance measurement at 850 nm was performed using a custom-built, low-cost BPW83 photodiode system. The Projectina SP-2000 spectrometer was operated in absorbance mode (spectral resolution 8 nm), and spectra were baseline-corrected against an unprinted polyester reference.

**Figure 4:**
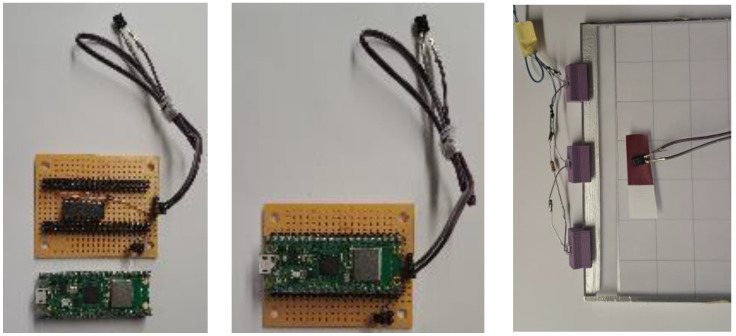
Low-cost BPW83 photodiode-based absorbance measurement system at 850 nm.

The BPW83 photodiode (Vishay Intertechnology, Inc., Malvern, PA, USA) is manufactured with an optical bandpass filter for 750–1150 nm, with a maximum relative spectral sensitivity peak at 950 nm. The signal is amplified using a low-noise transimpedance amplifier using Microchip MCP604 quad operational amplifier (op amp). The BPW83 converts incident infrared light into a photocurrent proportional to the optical intensity, which is then fed into the MCP604 configured as a transimpedance stage that converts this small current into a measurable output voltage. This voltage is subsequently read by the Pico’s ADC input, enabling calculation of both the corresponding voltage and the derived photocurrent. Calibration was performed by measuring and amplifying the reverse light current on the BPW83 without the polyester sample and then with the unprinted and printed polyester sample at the same distance from the LEDs, as shown in Figure 4 (right). Spectral absorbance curves from both methods were compared to validate the BPW83 low-cost photodiode approach against laboratory instrumentation, as it can be used in the field for measuring absorbance at 850 nm.

### 2.5. NIR Image Acquisition and Detection

**Figure 5:**
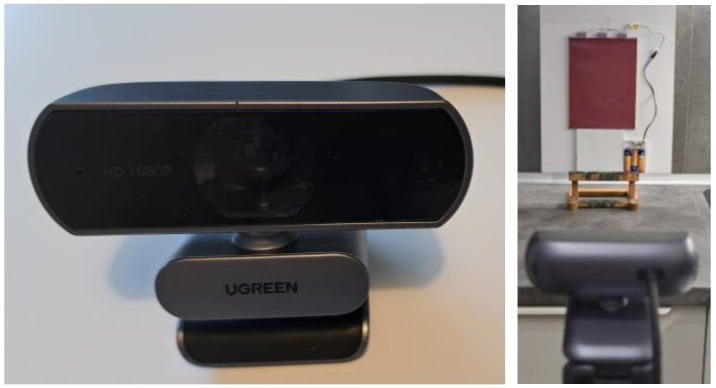
Modified UGreen USB camera (resolution of 1920 × 1080), with a bandpass filter from 700 to 1000 nm range (**left**) and distance measurement setup (**right**).

In this study, a low-cost UGreen USB camera (Ugreen Group Limited, Shenzhen, China) with a resolution of 1920 × 1080 px was used, as shown in Figure 5. The camera was adapted for the IR range by removing the built-in filter that blocks the NIR spectrum and installing a 0.2 mm thick filter foil that passes light in the 700–1000 nm range while blocking the visible spectrum (400–700 nm).

**Figure 6:**
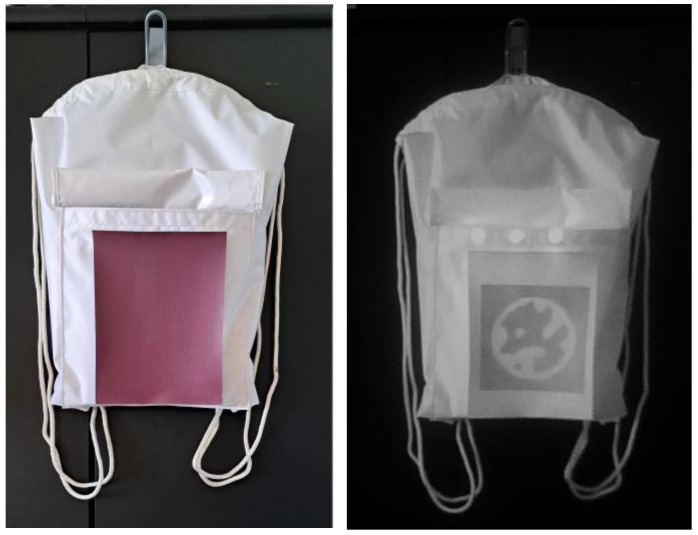
Polyester prototype bag with printed hidden STag code, shown in daylight in the visible spectrum (**left**) and in the NIR spectrum (**right**).

The NIR visibility of the patterns was evaluated using a camera without an IR-cut filter and with a NIR bandpass filter sensitive to the 700–1000 nm range. The camera operated in passive detection mode with no integrated NIR illumination, relying solely on the active planar illumination from the PMMA light guide. Camera parameters were manually set to maximize contrast. Captured images were processed in real-time using the OpenCV and STag marker detection library to identify and locate the NIR printed codes, as shown in Figure 6.

**Figure 7:**
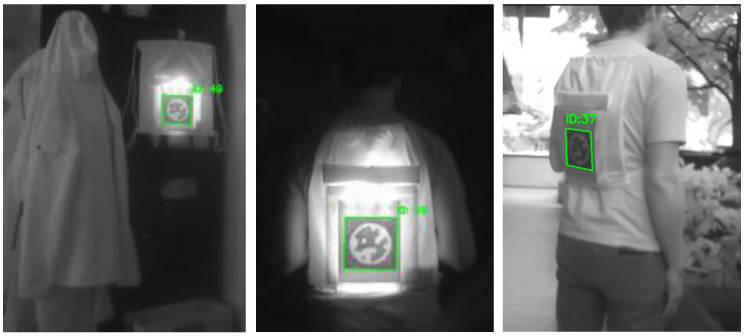
Polyester prototype bag with printed hidden STag code, shown in the NIR spectrum at different distances and angles ((**left**), (**middle**), and (**right**)).

Detection of the polyester textile in complete darkness and in daylight using the UGreen USB camera is shown in Figure 7, where the printed STag marker is visibly detectable.

The proposed method of integrating IR LEDs provided excellent visibility of the STag marker. We observed a more precise detection of the marker on the polyester bag in darkness with the provided pipeline in a controlled environment. In daylight conditions, the object remains detectable regardless of whether the IR LED is on or off, as illustrated in Figure 7 (right image).

### 2.6. Data Acquisition and Real-Time Camera-Based Detection Pipeline

NIR camera detection was quantified by measuring the distance at which STag code detection occurred in dark conditions under a controlled NIR illumination. Detection range tests were conducted by increasing the camera-to-sample distance in 50 cm increments until reliable code recognition dropped below 10% of the weighted average detection rate. The real-time acquisition and STag detection pipeline was implemented in Python (version 3.13.5), using OpenCV (opencv-python version 4.12.0.88) for image I/O/processing and the STag library for fiducial recognition. Camera control was managed via OpenCV’s VideoCapture object, with manual overrides for exposure, gain, and gamma; all automatic functions (auto-exposure/white balance/focus) were disabled. Experiments were conducted using an exposure setting of −6.0 (UVC log_2_ s read-back), with gain set to 0.0 and unsharp masking enabled. Pre-processing parameters were kept constant throughout each run.

To support quantitative reporting, the measurement script logs per-frame detections, and for each 60-frame block (~6 s at 10 fps) computes the pooled (weighted) detection rate with Wilson 95% confidence intervals and the mean tag edge lengths (px).

## 3. Results

### 3.1. Spectroscopy of Twin Dyes

**Figure 8:**
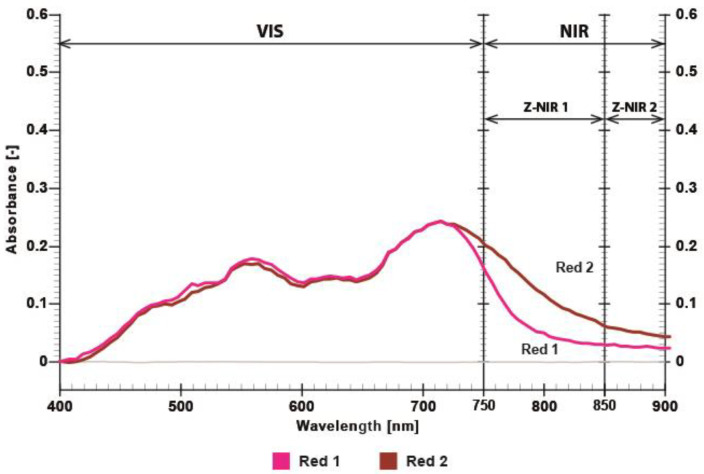
Absorbance of printed twin dyes, where Red 1 and Red 2 differ in NIR spectrum.

Three different NIR-absorbing twin dye formulations (coded as Red 1/Red 2, Blue 1/Blue 2, and Olive 1/Olive 2) were digitally defined using CMYK color values and printed as STag markers with dimensions of 120 × 120 mm. All other printing parameters (resolution, ink layer thickness, and curing profile) were kept constant. The objective was to investigate the effect of color formulation on absorption characteristics and the STag detection via the NIR camera. Dye twins in red, blue, and olive shades were produced, and spectrophotometric curves of the printed twin dyes were obtained through spectroscopic measurements. The first dye (Red 1—CMYK value: 43, 100, 47, 15) has low absorption in the NIR part of the spectrum, while the second dye (Red 2—CMYK value: 7, 92, 14, 40) exhibits strong absorption detectable by the NIR camera, as shown in Figure 8. Spectral absorbance measurements of the red twin dye printed on polyester samples (via DTF transfer) revealed two distinct absorption features: one corresponding to absorptions values in the visible range with a peak near 700 nm that is almost identical, and different absorption values in the NIR range between 750 and 900 nm.

**Figure 9:**
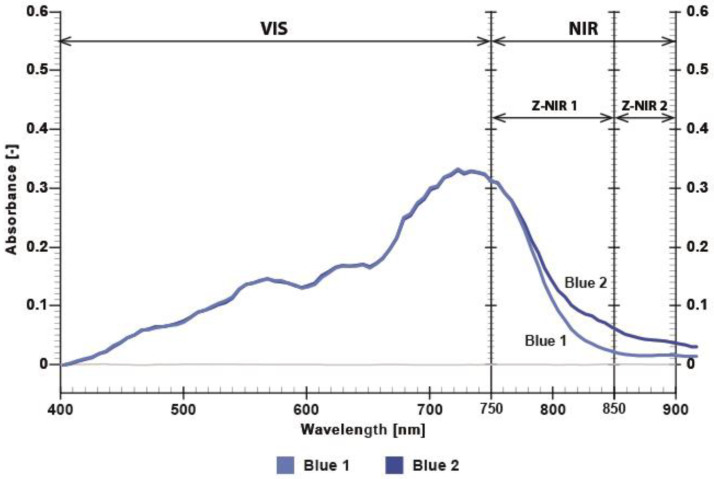
Absorbance of printed twin dyes, where Blue 1 and Blue 2 differ in NIR spectrum.

In the NIR range (750–900 nm referred to as Z-NIR), the printed regions exhibited a maximum absorbance difference ΔZ (the absorbance difference between the two dyes in the NIR range) [23]. The two red dyes (Red 1 and Red 2) exhibited an absorbance difference of approximately 0.05 absorbance units (A.U.) around 850 nm (Figure 8). Absorbance of the blue twin dyes (Blue 1—CMYK value: 100, 30, 24, 0 and Blue 2—CMYK value: 96, 4, 4, 22) is shown on Figure 9. The two blue dyes exhibited an absorbance difference of approximately 0.04 A.U. at around 850 nm (Figure 9).

**Figure 10:**
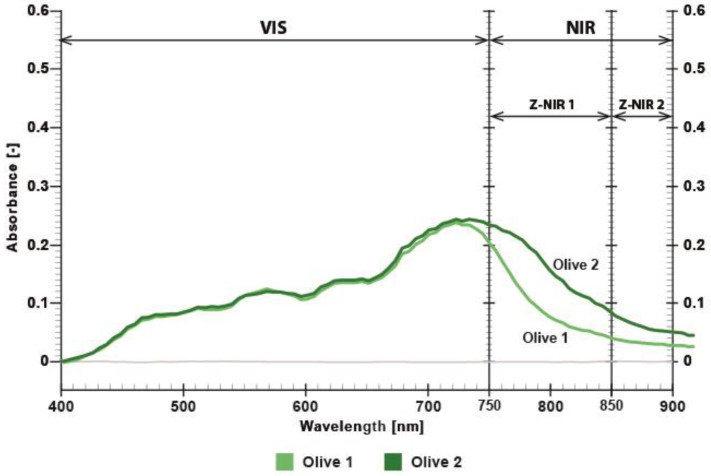
Absorbance of printed twin dyes, where Olive 1 and Olive 2 differ in the NIR spectrum.

Figure 9 shows that the absorption values of blue dyes in the visible range are identical up to 750 nm, indicating that these dyes cannot be distinguished in the visible part of the spectrum. Figure 10 shows that the olive dyes (Olive 1—CMYK value: 34, 36, 100, 15 and Olive 2—CMYK value 3, 3, 100, 40) are almost identical in the visible range up to 720 nm and exhibit an absorbance difference of approximately 0.04 A.U. at 850 nm.

This difference represents the contrast of the STag code at the target wavelength, which can be measured by the BPW83 photodiode setup and that can be detected and decoded by the OpenCV/STag software (opencv-python version 4.12.0.88).

### 3.2. Photodiode-Based Measurements

**Figure 11:**
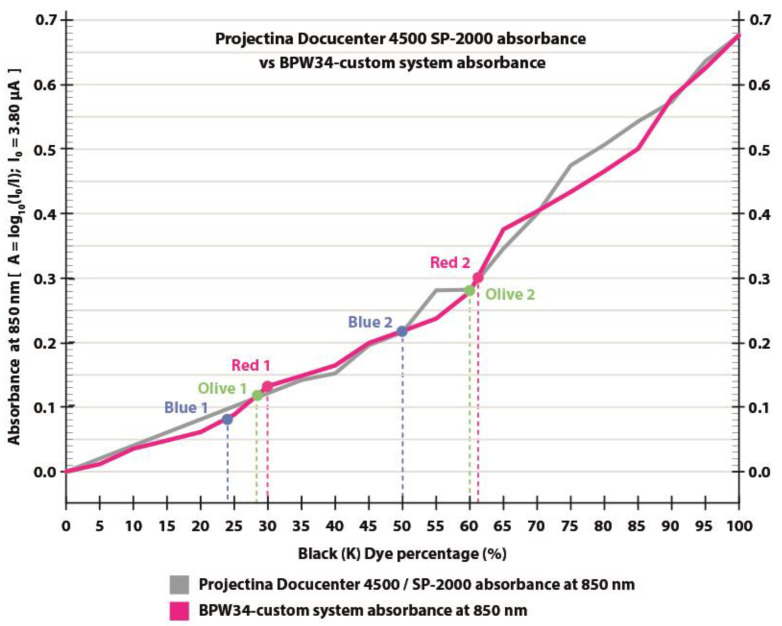
Comparison of NIR absorbance at 850 nm measured with a low-cost photodiode (BPW83, 750–1050 nm bandpass, and constant IR-LED) and laboratory spectroscopy (Projectina Docucenter 4500 SP-2000, 8 nm resolution). Curves show absorbance vs. printed black coverage (0–100%), computed as *A = log_10_ (I_0_/I_sample_)* for BPW83 system.

The BPW83 photodiode setup with a 750–1050 nm bandpass filter confirmed the absorbance trend, showing close agreement with the Projectina Docucenter 4500 SP-2000 spectroscopy at 850 nm under constant IR LED illumination (Figure 11). Measurements were performed on printed patches of AZON black dye from 0% (white) to 100% (black) in 5% increments.

The BPW83 photodiode system was calibrated and validated prior to all absorbance measurements to ensure accuracy and reproducibility. Calibration was executed in three stages under constant IR LED illumination (3.3 V supply with a 100-ohm series resistor) and a fixed geometry with a 30 mm distance between the LED source and the photodiode. First, a reference signal was acquired (*I_ref_*) to determine the maximum photocurrent output without attenuation. Second, the polyester substrate without any print (*I_0_*) was measured to account for substrate-specific absorption and scattering. Finally, the printed sample (*I_sample_*) was positioned with the printed side facing the sensor, and the transmitted light was recorded. Absorbance (*A*) was calculated using the standard Beer–Lambert relation: *A = log_10_ (I_0_/I_sample_)*, where *I_0_* is the photocurrent measured through the unprinted polyester substrate, and *I_sample_* is the current transmitted through the printed patch. The photodiode current was derived from the measured voltage across a known load resistor and converted into optical power using the known responsivity of the BPW83 photodiode (0.62 A/W). To assess measurement reliability, each data point was measured three times for all dye coverage levels from 0% to 100% in 5% increments, as well as for the reference and substrate. The standard deviation of the measured photocurrent across the three repetitions was generally below ±0.5 µA, corresponding to a relative standard deviation (RSD) of less than 2% for most measurements. The highest RSD values (up to ~5%) were observed near 50% coverage and in the highly absorbing region (>70% black), which is attributed to ADC quantization noise and small fluctuations in the LED output. Error propagation analysis of the absorbance calculation showed that the combined measurement uncertainty is dominated by the photocurrent noise term.

Using the propagated error of absorbance formula ΔA ≈ (1/ln(10)) * sqrt((ΔI_0_/I_0_)^2^ + (ΔI_sample_/I_sample_)^2^), the estimated absorbance uncertainty was ±0.01 A.U. for most measurements, increasing slightly to ±0.02 A.U. for samples with very low transmitted intensity. The main sources of measurement uncertainty are photodiode and ADC noise (±0.3–0.6 µA) arising from electronic noise and signal digitization, LED output fluctuation (<1%) due to minor variations in light intensity, sensor alignment and distance tolerance (<1%) related to the possible geometrical misalignment of the setup, photodiode responsivity tolerance (±5%) specified by the manufacturer affecting absolute calibration, and baseline calibration error (±0.3–0.5 µA) originating from variations in the three repeated measurements of the unprinted substrate samples.

Despite these uncertainties, the absorbance values obtained with the BPW83 system showed excellent agreement with the reference spectrometer (Projectina SP-2000), with a correlation coefficient of R^2^ ≈ 0.98 validating the low-cost sensor approach. The Projectina SP-2000 absorbance values were normalized to BPW83 photodiode setup measurements using the regression Formula (1):

SP-2000 _normalized_ ≈ a + b * BPW83 _measured_absorbance_
 with parameters: a = −0.2628                             b = 1.9097(1)

The absorbance of red, blue, and olive dye pairs, as shown in Figure 11, was measured using a BPW83 photodiode. The difference in absorbance at 850 nm between the two red and the two olive samples is slightly greater than 30%, while the blue dye pair exhibits a difference of approximately 26%.

### 3.3. Colorimetric Analysis

Color difference (CIE 2000 L*a*b* ΔE) [24] between the twin dyes was evaluated using X-Rite eXact device under D50 2^0^ illumination in the visible spectrum. The twin dyes Red 1 and Red 2 showed a color difference of ΔE* = 2.06, which is visually indistinguishable to the human eye. The absorbance difference (ΔZ) in the NIR range for the red twin dye measured from the spectrograph, shown in Figure 8, is 0.05 A.U. at 850 nm. This confirms that the red twin dyes are visually unobtrusive while still producing strong NIR absorbance, as shown in Figure 11. Table 1 shows the CMYK recipes of twin dyes and measured CIE L*a*b* values with calculated CIE 2000 L*a*b* ΔE. The blue and olive dye have very small color differences, as can be seen in the visible part of the spectrum in Figure 9 and Figure 10.

**Figure 12:**
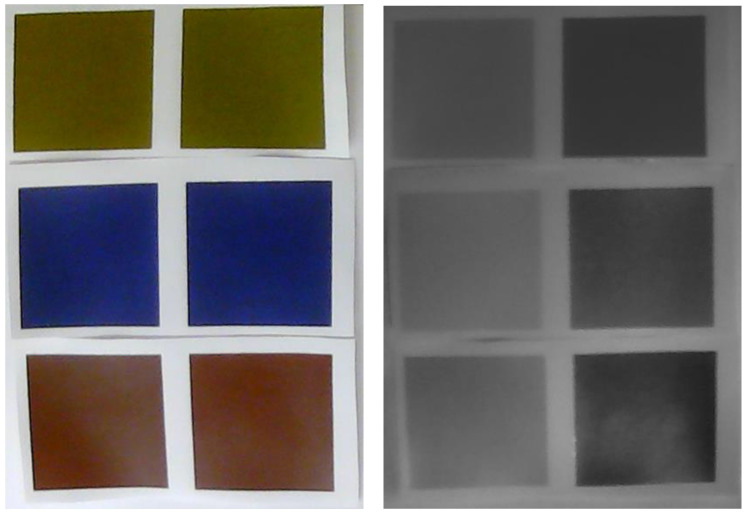
Twin dye samples shown in the visible spectrum (**left**) and the NIR spectrum (**right**).

Figure 12 shows the samples captured in the visible and near-infrared ranges, using an unmodified and a modified webcam, respectively.

While the twin dyes exhibit very small color differences in the visible part of the spectrum, they introduce a significant difference in contrast in the near-infrared part of the spectrum, as seen in Figure 12. The BPW83 photodiode operates as a single-element detector with a built-in optical filter that passes infrared radiation between approximately 750 and 1150 nm, peaking near 950 nm. It therefore measures the absorbance over a relatively narrow spectral window. In contrast, the CCD camera used in this study, equipped with an external filter that blocks visible light (400–700 nm) but transmits NIR radiation (700–1000 nm), integrates reflected light across the entire near-infrared range. As a result, the camera captures a much stronger overall contrast between the twin dyes, since it effectively perceives the integral of the NIR absorbance spectrum, while the BPW83 detects only a fraction of it.

### 3.4. Detection Range Testing

The NIR camera-based recording system was tested in a controlled environment with external planar illumination, measuring detection success over distance, as shown in Table 2.

Each distance aggregates 8 × 6 s blocks at 10 fps (n = 480 frames). Values are pooled (weighted) detection rates with Wilson 95% confidence intervals; code dimension is reported as the mean edge length in pixels.

Using an NIR camera with external planar backlighting and normal incidence, the pooled per-frame detection rate decreased with distance from 86.25% (95% CI 82.88–89.04) at 1.0 m to 63.12% (58.72–67.32) at 1.5 m and 26.67% (22.91–30.80), 14.79% (11.90–18.25), and 8.12% (6.00–10.91) at 2.0, 2.5, and 3.0 m, respectively. The decline parallels the reduction in the rendered code dimension from 105 × 105 px at 1.0 m to 39 × 39 px at 3.0 m, with a marked loss of reliability once the tag falls below ~50–60 px per side.

Each distance aggregates eight 6 s windows at 10 fps (n = 480 frames). The intervals reported are Wilson 95% CIs computed on pooled detections, indicating stable estimates at this sampling depth. With fixed camera settings (exposure: −6.0, gain: 0.0, and unsharp: on), the data indicate that reliable detection (>60%) is sustained up to about 1.5 m, whereas performance beyond 2.0 m is limited for the selected low-cost UGreen camera and optics used in the camera.

### 3.5. TIR Illumination Uniformity

**Figure 13:**
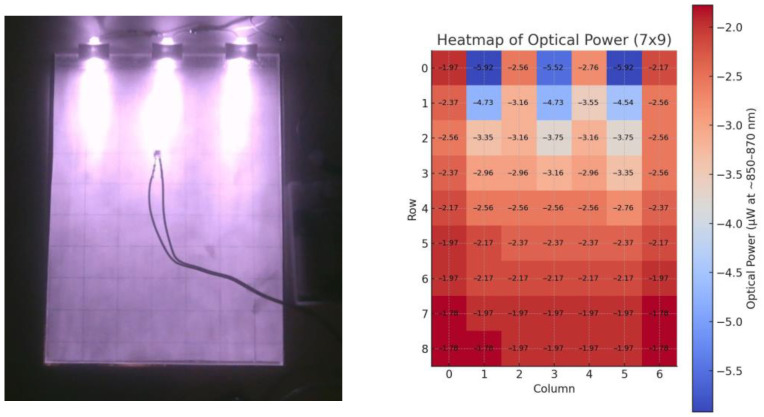
Illumination setup with measurement pattern of PMMA plexiglass TIR (**left**) and heatmap analysis (**right**).

The total internal reflection (TIR) illumination system was evaluated to determine the spatial uniformity of NIR irradiance across the 15 × 20 cm plexiglass light guide. Illumination intensity was mapped using the BPW83 photodiode at 850 nm over a 7 × 9 grid (63 measurement points), with each value recorded under a constant LED drive current (18.1 mA, 4.5 V). The measured photocurrent ranged from 0.9 µA to 3.0 µA, corresponding to an optical power between 1.8 µW and 5.9 µW, assuming a responsivity of 0.507 A/W at 870 nm. Across the entire illuminated area, the relative deviation from the mean intensity was approximately +120%/−34%, with the central region remaining within ±10% of the mean value. The highest intensities were observed near the upper section of the grid, while the peripheral areas exhibited lower reflected power (Figure 13).

This non-uniform illumination configuration was intentionally adopted to test a low-cost, do-it-yourself (DIY) approach for creating an illuminated detection platform using only three infrared LEDs. Despite the simplified geometry, the illumination uniformity achieved was sufficient to ensure stable marker detection under dark-field conditions. The small spatial variation in intensity at the center of the plane did not significantly affect STag recognition performance, as confirmed by consistent detection rates up to 1.5 m in the range tests. The heatmap analysis (Figure 13, right) visualizes the measured photocurrent distribution and confirms that illumination unevenness within 10% does not critically influence detection reliability for compact planar surfaces. Nevertheless, the future optimization of LED arrangement, diffuser surface treatment, and edge-coupling geometry is planned to further improve irradiance uniformity, especially for larger-scale or multi-marker configurations. These improvements will enhance reproducibility and support scaling of the system toward commercial smart-packaging or multi-object detection applications.

### 3.6. Summary of Findings

Twin dye printing on polyester produced strong and quantifiable NIR contrast (ΔZ = 0.04–0.05 A.U. at 850 nm) while remaining visually unobtrusive in the visible spectrum (ΔE_00_ < 2.1), enabling covert STag encoding on textile substrates. The evaluation of three twin dye color pairs (red, blue, and olive) confirmed that these spectrally matched formulations provide a stable optical performance across different hue families. The low-cost BPW83 photodiode readout showed agreement with the laboratory-grade spectrometer at 850 nm, achieving a correlation coefficient of R^2^ ≈ 0.98 and a measurement uncertainty below ±0.02 absorbance units, which validates the feasibility of field measurements without the need for benchtop instrumentation.

Under planar total internal reflection (TIR) back-illumination, the weighted per-frame detection rate remained above 60% up to ~1.5 m, decreasing progressively beyond 2.0 m as the rendered tag size dropped below 50–60 pixels per side. These results demonstrate that detection reliability is primarily governed by the optical resolution and uniformity of illumination rather than dye performance.

Together, these findings confirm the viability of a low-cost, self-illuminated NIR tagging approach that combines printable twin dye materials, simple optoelectronic components, and open-source computer vision algorithms to achieve reliable and reproducible code detection in complete darkness. This integration bridges laboratory spectroscopy and field-deployable sensing, offering a scalable solution for smart packaging, covert identification, and autonomous visual systems operating in low-light or unstructured environments.

## 4. Discussion and Conclusions

This study presents a novel and cost-effective approach for integrating near-infrared (NIR) absorbent twin dyes into polyester packaging for use as machine-readable identification tags. By combining laboratory-grade NIR spectroscopy with low-cost photodiode-based absorbance measurement, we have demonstrated that it is possible to accurately characterize the spectral response of dyed polyester materials and correlate it with real-world detection performance across several twin dye color pairs. The dual measurement methodology ensures that both precise optical characterization and practical field readiness are evaluated, with results confirming a strong correlation (R^2^ ≈ 0.98) and measurement uncertainty below ±0.02 A.U.

Unlike conventional NIR tagging systems that rely on the camera’s own active illumination, the proposed method incorporates edge-coupled infrared LEDs directly into the package structure, providing self-illumination and ensuring reliable detection in complete darkness or low-light conditions (e.g., nighttime outdoor environments, warehouses, and unlit transport hubs). By eliminating the need for camera-mounted NIR illuminators, the system reduces the complexity, power consumption (<0.1 W), and cost (<50 EUR), while maintaining portability and autonomy (mass < 350 g).

Our results indicate that the use of twin dyes enables enhanced contrast in the NIR spectrum (ΔZ = 0.04–0.05 A.U. at 850 nm) while remaining visually unobtrusive in the visible range (ΔE_00_ < 2.1). Measurements confirmed stable absorbance peaks at 850 nm and reproducibility with a relative standard deviation below 2%. Detection performance remained >60% up to 1.5 m and declined beyond 2.0 m, primarily due to image resolution limits. These findings demonstrate the feasibility of low-cost NIR identification systems for smart packaging, security marking, and covert logistics tracking, particularly in scenarios where ambient lighting cannot be guaranteed.

The study also acknowledges limitations regarding durability, environmental stability, and outdoor robustness. Although polyester substrates and pigment-based inks exhibit strong lightfastness (ISO 105 B02 ≥ 6 [25]), extended testing under temperature variation, UV exposure, and mechanical stress remains necessary. Future research will therefore focus on optimizing illumination geometry for improved uniformity, testing new dye formulations for higher contrast, and extending environmental and durability trials. Planned developments include the use of high-resolution 4K cameras with adaptive exposure control and AI-assisted image enhancement to improve detection reliability, extend the operational range, and establish a more comprehensive understanding of system stability and reproducibility.

## Figures and Tables

**Table 1:** Twin dyes color recipes and color difference measurements.

Twin Dye Name	CMYK of the 1st Dye	CMYK of the 2nd Dye	CIE L*a*b* of the 1st Dye	CIE L*a*b* of the 2nd Dye	CIE 2000 L*a*b* ΔE
Red	43, 100, 47, 15	7, 92, 14, 40	33.14, 41.07, 11.45	30.53, 41.10, 11.38	2.06
Blue	100, 30, 24, 0	96, 4, 4, 22	24.62, −1.54, −35.30	24.73, −1.70, −33.44	0.75
Olive	34, 36, 100, 15	3, 3, 100, 40	40.87, −3.45, 30.38	41.21, −3.83, 31.81	1.33

**Table 2:** Detection rate of STag on polyester (red twin dyes) versus distance under NIR backlit illumination with an NIR camera.

Distance (m)	Detections Total (k)	Weighted Average Detection Rate (%)	95% CI Low (%)	95% CI High (%)	Code Dimension (pixels) at Recorded Distance
1.0	414	86.25	82.88	89.04	105 × 105 px
1.5	303	63.12	58.72	67.32	72 × 72 px
2.0	128	26.67	22.91	30.80	54 × 54 px
2.5	71	14.79	11.90	18.25	46 × 46 px
3.0	39	8.13	6.00	10.91	39 × 39 px

## Data Availability

Data are contained within the article.
